# Debunking the Myth of the Specialty Soulmate

**DOI:** 10.1007/s40670-026-02689-5

**Published:** 2026-03-04

**Authors:** Anita Pokorny, Nicole J. Borges

**Affiliations:** 1 Department of Medical Education, Geisel School of Medicine at Dartmouth, Hanover, NH Germany; 2 Family and Community Medicine at Northeast Ohio Medical University, Rootstown, Ohio USA

**Keywords:** Vocational theory, Specialty choice, Career advising, Undergraduate medical education

## Abstract

Medical students are commonly advised to “find their fit” as they strive to make a specialty decision. But the term “fit” is often interpreted to mean that every medical student has a specialty soulmate, one perfect specialty that will provide career satisfaction. The specialty soulmate myth is reinforced through the career advising process, the residency application and selection process, and the structure and funding of graduate medical education. The authors explain the vocational theory that is the foundation of fit, describe how the specialty soulmate myth is reinforced in undergraduate and graduate medical education, and offer recommendations for dispelling the specialty soulmate myth.

Medical students are commonly advised to “find their fit” as they strive to make a specialty decision. But the term “fit” is often interpreted to mean that every medical student has a specialty soulmate, one perfect specialty that will provide career satisfaction. The specialty soulmate myth is reinforced through the career advising process, the residency application and selection process, and the structure and funding of graduate medical education.

## The Foundation of Fit

The term fit comes from vocational psychology’s theory of person-environment fit [1] which shares similar or equivalent concepts with other theories prominently recognized across academic fields [2, 3]. As such, it is widely recognized that individuals who fit their field, work setting/environment, and other related factors are happier and more satisfied and, thus, more engaged, productive, and/or high performing.

Matching interests and skills to the content of the specialty is probably the most common understanding of fit. But fit also means integrating work with other priorities such as family, friends, hobbies, and civic engagement [4]. Finally, fit means seeking opportunities that are personally meaningful and rewarding, based on values, interests, and beliefs [5, 6]. These different aspects of fit are not mutually exclusive. In this context, fit is multifaceted and complex, and finding fit is an active and life-long process that requires research, observation, and reflection [7].

## Reinforcing the Specialty Soulmate Myth

Current career advising strategies primarily focus on matching skills and abilities to a single specialty, presenting this as the crux of a student’s specialty decision and reinforcing the myth of the specialty soulmate. Although medical students begin to shift their considerations about specialty fit from factors like work enjoyment to lifestyle factors, such as schedule and hours worked as they progress in their medical training [8], this aspect of fit is often minimized or dismissed. Current advising strategies also leave out the concept of values-based career decision-making, which is a crucial element of fit. This aspect of fit can be difficult to grasp, so is often overlooked or ignored when advising a student on specialty choice.

The residency application and matching process also implies that medical students have a specialty soul mate. Although students are commonly encouraged by medical school career advisors to consider alternative career paths in case their “ideal” doesn’t pan out, referring to them as back up or parallel plans implies that a student who fails to match with their specialty soulmate must settle for a lesser option. The residency selection process also penalizes students for their forethought in creating alternative pathways, often requiring them to hide their application to an alternative specialty.

Finally, the structure of Undergraduate Medical Education-Graduation Medical Education (UME-GME) is hard wired to reinforce the specialty soulmate myth by creating policy barriers that make it difficult for physicians to change specialties during residency and after. The funding mechanism for Graduate Medical Education [9] places pressure on unmatched candidates to choose a specialty path out of desperation and makes it difficult for resident physicians to change specialties if they find they are on the wrong path.

## Recommendations

To imply that there is only one perfect specialty for each physician puts undue stress on medical students and can lead to dissatisfaction and disillusionment for those who don’t achieve their perceived “ideal” [10]. The following actions can aid in dispelling the specialty soulmate myth:

- Educate general and specialty career advisors about foundational career theory, so they are better equipped to help medical students find their specialty fit.
- Dedicate curriculum hours during each year of medical school to required career advising activities and encourage the use of a reflective learning portfolio that can assist students in making important connections between lived experiences and enable values-driven career decisions.
- Reimagine the “parallel plan” as an alternative pathway for creating a meaningful career and reframe residency as the next step in training, not a rigid path from which there is no deviation.
- Normalize selection of a general intern year (PGY1 only) as a means for undecided medical students to make informed decisions about graduate medical education.
- Advocate for changing the funding structure of graduate medical education so that the Initial Residency Period (IRP) is calculated starting with the second year of postgraduate training. This will take the pressure off unmatched students, who represented approximately 20% of participants in the 2025 National Residency Matching Program (NRMP) [11], and provide flexibility for other residents to switch specialties after the first year of residency training. This is consistent with recommendations of the Coalition for Physician Accountability Undergraduate Medical Education-Graduation Medical Education Committee (UGRC) [12].

## Conclusions

Debunking the myth of the specialty soulmate requires a major paradigm shift throughout Undergraduate and Graduate Medical Education. Ensuring that all medical students have the tools and resources needed to make educated career decisions, with guidance from well-trained career and specialty advisors, is the foundation for this change. In addition, creating pathways for undecided and unmatched medical students to continue exploring career options without stigma or systemic barriers can lead to a happier and healthier physician workforce.
